# Ensuring fidelity: key elements to consider in disseminating a diabetes telemanagement program for underserved Hispanic/Latinos living with type 2 diabetes

**DOI:** 10.3389/fcdhc.2024.1328993

**Published:** 2024-02-16

**Authors:** Sabrina Martinez, Christian N. Nouryan, Myia S. Williams, Vidhi H. Patel, Paulina Barbero, Valeria Correa Gomez, Jose Marino, Nicole Goris, Edgardo Cigaran, Dilcia Granville, Lawrence F. Murray, Yael T. Harris, Alyson Myers, Josephine Guzman, Amgad N. Makaryus, Samy I. McFarlane, Roman Zeltser, Maria Pena, Cristina Sison, Martin L. Lesser, Myriam Kline, Ralph Joseph DiClemente, Renee Pekmezaris

**Affiliations:** ^1^ Northwell Health System, Department of Medicine, Manhasset, NY, United States; ^2^ Zucker School of Medicine at Hofstra Northwell, Department of Medicine, Hempstead, NY, United States; ^3^ Feinstein Institutes for Medical Research, Manhasset, NY, United States; ^4^ Nassau University Medical Center, Department of Medicine, Uniondale, NY, United States; ^5^ SUNY Downstate Health Sciences University, Department of Medicine, Brooklyn, NY, United States; ^6^ Mount Sinai Hospital, Department of Medicine, NY, Rego Park, NY, United States; ^7^ New York University, School of Global Public Health, New York, NY, United States

**Keywords:** diabetes, Hispanic, Latino, underserved, telemanagement, bilingual, self-management

## Abstract

**Background:**

The Hispanic/Latino population has greater risk (estimated >50%) of developing type 2 diabetes (T2D) and developing it at a younger age. The American Diabetes Association estimates costs of diagnosed diabetes in 2017 was $327 billion; with medical costs 2.3x higher than patients without diabetes. The purpose of this manuscript is to describe the methodology utilized in a randomized controlled trial aimed at evaluating the efficacy of a diabetes telemanagement (DTM) program for Hispanic/Latino patients with T2D. The intent is to provide information for future investigators to ensure that this study can be accurately replicated.

**Methods:**

This study was a randomized controlled trial with 240 participants. Eligible patients (Hispanic/Latino, aged 18+, living with T2D) were randomized to Comprehensive Outpatient Management (COM) or DTM. DTM was comprised of usual care, including routine clinic visits every three months, as well as: Biometrics (a tablet, blood glucose meter, blood pressure monitor, and scale); Weekly Video Visits (facilitated in the patient’s preferred language); and Educational Videos (including culturally congruent diabetes self-management education and quizzes). COM consisted of usual care including routine clinic visits every three months. For this study, COM patients received a glucometer, glucose test strips, and lancets. Establishing a therapeutic nurse-patient relationship was a fundamental component of our study for both groups. First contact (post-enrollment) centered on ensuring that patients and caregivers understood the program, building trust and rapport, creating a non-judgmental environment, determining language preference, and establishing scheduling availability (including evenings and weekends). DTM were provided with a tablet which allowed for self-paced education through videos and weekly video visits. The research team and Community Advisory Board identified appropriate educational video content, which was incorporated in diabetes educational topics. Video visits allowed us to assess patient involvement, motivation, and nonverbal communication. Communicating in Spanish, and awareness of diverse Hispanic/Latino backgrounds was critical, as using relevant and commonly-used terms can increase adherence and improve outcomes. Shared decision-making was encouraged to make realistic health care choices.

**Conclusion:**

Key elements discussed above provide a framework for future dissemination of an evidence-based DTM intervention to meet the needs of underserved Hispanic/Latino people living with T2D.

## Introduction

As the prevalence of Type 2 Diabetes Mellitus (T2DM) among Hispanic/Latino people living in the United States (US) continues to rise, it is absolutely critical to implement effective strategies to improve patient outcomes and reduce cost of treatment (1). The US Census Bureau (2) estimates that the Hispanic/Latino population will increase to 111 million by 2060, compared to 62.3 million in 2020. According to the Centers of Disease Control and Prevention (CDC), Hispanic/Latino people are more likely to have T2DM (17%) compared to non-Hispanic/Latino White people (8%).

The risk of developing T2DM in the US Hispanic/Latino population is estimated to be greater than 50%, and there is a greater risk of developing T2DM at a younger age (3). Several risk factors reported by Aguayo-Mazzucato et al. (4) contribute to this including sociocultural (low income and less access to education and health care), and a genetic predisposition of obesity and high resistance to insulin.

Schneiderman et al. (5) reported that only 48% of Hispanic/Latinos in the Hispanic Community Health Study/Study of Latinos had adequate glycemic control (defined as an HbA1c of <7%). According to the CDC, Hispanic/Latino Americans also suffer from higher rates of complications associated with T2DM such as kidney failure, vision loss, and blindness. This indicates an urgent need for implementation of evidence-based interventions that will increase access to care and improve health care quality and patient centered outcomes, especially among underserved Hispanic/Latino Americans (6).

The literature supports the effectiveness of telemedicine/telehealth on glycemic control, self-efficacy, and self-care in patients living with T2DM. In 2021, a systematic review by 7, based on a quantitative synthesis of 43 studies, found telemedicine provided by videoconference or interactive telephone significantly reduced HbA1c for patients living with T2DM. Zhang et al. (8) performed a meta-analysis of 32 articles, assessing the effectiveness of telemedicine interventions in primary care for the management of patients living with T2DM, and found reductions in HbA1c, fasting glucose, and postprandial glucose. A randomized controlled trial (RCT) of 200 participants (9); and a qualitative study (10); showed that a comprehensive telehealth intervention improved HbA1c, diabetes distress, diabetes self-care, and self-efficacy at a reasonable cost in patients with persistently poorly controlled T2DM. Another prospective RCT by Warren et al. (11); showed that participants that received the telehealth intervention had a reduction in HbA1c, and total healthcare costs, including intervention costs, were lower compared with usual care.

Although the literature supports innovative interventions to improve outcomes in patients living with T2DM, treatment credibility and consistency is important to show the proposed intervention is strongly connected to patient centered outcomes (12, 13). When implementing evidence-based interventions in clinical practice, any deviation from PICOTS (population, intervention, comparators, outcomes, time, and setting) may have an impact on the efficacy of the results (14, 15). As stated by Keith et al. (16): “fidelity of an intervention’s implementation reflects how an intervention is, or is not, used in clinical practice and is an important factor in understanding intervention effectiveness and in replicating the intervention in dissemination efforts.” Adherence to key elements of a program design is imperative to ensure replication, validity, and reliability (17).

The goal of the telehealth intervention in the main study was to complement the traditional standard of medical care in diabetes to improve access to health care services and patient outcomes for underserved Hispanic/Latino people living with T2DM. The hypothesis was that patients randomized to receive telehealth would have improved glycemic management (main outcome), diabetes-related quality of life, blood pressure, medication adherence, and diabetes-related self-efficacy.

## Current study

The aim of this sub-study is to summarize the specific methodologies implemented for the Diabetes Telemanagement intervention used in a randomized controlled trial of underserved Hispanic/Latino participants. We seek to discuss key elements of a telemanagement program which were identified through a rigorous collaborative effort between the research team and a Community Advisory Board (CAB).

## Methods

The Patient and Caregiver-Centered Diabetes Telemanagement Program for Hispanic/Latino Patients was a four year (2019-2023) multiphase mixed method, RCT of 240 community-dwelling women (n=157) and men (n=83) with an average age of 55.7 (range: 21 to 88) old living in their homes at enrollment. The majority (57%) reported living with diabetes for over five years and many (23%) were uninsured. Eligible patients (over age 18, living with T2D, and being seen in one of nine clinics participating in the study) who consented to participate after being approached by our bilingual team of enrollers were randomized by our biostatistics department to Comprehensive Outpatient Management (COM) or COM plus Diabetes Telehealth Management (DTM). (ClinicalTrials.gov ID NCT03960424).

COM was based on 2018 ADA Standards of Medical Care in Diabetes, usually consisting of a review of past medical/family history, social history, medications, screening, physical examination, laboratory evaluation, etc., and routine visits every three months. For this study, patients randomized to COM also received a glucometer, glucose test strips, and lancets.

DTM was comprised of three components. For the first component (biometrics), patients receive a tablet and Bluetooth-enabled peripherals which included a blood glucose meter, blood pressure monitor, and weight scale, and were instructed how to use the components to measure their values for each component. Bluetooth technology enabled timely transmission of daily measures such as blood glucose, blood pressure, and weight to a clinical portal for daily review by the study nurse. Real time transmission of data allowed the nurse to intervene when readings were abnormal such as in hyperglycemia or hypoglycemia.

The second component (video visits) was a weekly telehealth visit facilitated by a culturally congruent registered nurse certified in diabetes care and education (CDCES). Weekly visits were conducted in the patients preferred language (English or Spanish).

The third component (educational videos and quizzes) was access to culturally congruent diabetes self-management educational videos and quizzes, also discussed with the nurse during the weekly telehealth visits. Answers to the quizzes were reviewed by the nurse and used to identify, address and close knowledge gaps.

## Theoretical framework

In this study, the Social Cognitive Theory (SCT) was used to promote change in health behaviors. SCT has been shown to improve health behaviors by altering cognitive processes and increasing an individual’s belief in their ability to accomplish tasks (18, 19). Sarkar et al. (20); showed that self-efficacy was significantly associated with diet, exercise, self-monitoring of blood glucose, and foot care.

During each telehealth visit, shared-decision making was encouraged which allowed us to collaboratively make optimal and realistic health care decisions. We routinely initiated the discussion on the measurements (blood glucose, blood pressure, and weight) for the previous week by asking patients to self-reflect on their performance. We asked questions such as “How do you think you did overall?” “What do you think was your biggest achievement this week?” “What did you improve on this week?” “What would you like to focus on this week?” “Do you believe you can do better, why or why not?”.

The answers to the questions were used to assess strengths and weaknesses and to facilitate discussions. Positive reinforcement was consistently used to encourage positive outcomes. Another key component of the weekly telehealth visit was to reiterate the importance of setting realistic short-term and long-term goals. Furthermore, Caregiver/family support and collaboration was constantly encouraged. Indeed, study patients reported that it was easier to adhere to healthy lifestyle changes when they had the support of their family as everyone shared the same interest and as a result the entire family made healthier choices such as staying physically active and eating healthier foods.

## Building rapport

Establishing a therapeutic nurse-patient relationship was a fundamental component of our study. Upon completion of the informed consent process, each patient was contacted by phone within 24-72 hours by the research nurse. The first phone contact (post-enrollment) was mainly centered on ensuring that the patient and caregiver (if applicable) had a thorough understanding of the program, as well as building rapport, creating a trusting and non-judgmental environment, and determining language preference. Both nurses in our study, along with the four recruiters who participated, spoke fluent Spanish and shared a Hispanic/Latino Heritage with the patients.

## Determining patient preferences for interaction

During first contact, our team addressed preferred language for communication and scheduling availability to accommodate the needs of our participants. Regardless of the language spoken when the call was answered, we routinely asked whether participants preferred to continue the call in English or Spanish. Although some patients spoke both languages, when asked about preference, many felt more comfortable speaking in Spanish. Often, patients reported that a complex topic such as diabetes management is easier to comprehend when education was provided in their preferred language.

In addition, we focused on scheduling the first and subsequent telehealth visits (as indicated) at a time that worked best for each individual patient. We provided early morning, evening, and weekend appointments to accommodate the needs of our patients with scheduling conflicts. Offering scheduling flexibility allowed patients to schedule visits at times that were most convenient for them which may have improved adherence to telehealth visits with the nurse.

Study participants often missed or rescheduled routine follow-up appointments which they attributed to having two or three jobs or a varying schedule. For example, patients who reported employment as construction workers, had a work schedule that varied daily, and consequently were unable to schedule or adhere to appointments during the traditional office appointment schedule – weekdays between 9:00 AM and 5:00 PM. Taking time off from work for a doctor/telehealth appointment was not an option for most patients.

## Technology training

While the literature supports the effectiveness of telehealth to improve glycemic control in patients with T2DM, telehealth may also be intimidating and underutilized in patients who are not well-versed in technology. To close gaps in knowledge, we provided self-paced education on how to use the tablet and Bluetooth-enabled peripherals. Training was initiated by the recruiters when they delivered the tablet to the patient’s home. Additional teaching was provided by the nurse during the weekly telehealth visits based on patient needs.

All telehealth patients had access to an educational video with step-by-step instructions on how to use the device in their preferred language (English or Spanish). Patients were able to watch the videos as often as they desired for the six month intervention. Written instructions were given to the patients in simple, easy to understand language and included images to build comprehension. All written communication was provided to the patients in their preferred language.

A previous lack of adequate training in glucometer use was common and was reported to have a negative impact on adherence. For example, some patients reported non-adherence to blood glucose monitoring as recommended by their provider due to the inability to use their personal blood glucose meter. Our team reported that patients were more likely to adhere to self-monitoring of blood glucose when they were appropriately educated on how to use a blood glucose meter. Ultimately, all patients that received training on how to use the devices reported feeling confident in the use of the equipment provided and were also more likely to adhere to daily monitoring, blood pressure and weight.

## Community input

The research team and CAB performed an extensive search of diabetes educational video content available online from multiple vetted sources. Based on CAB feedback, most of the content originally presented was found to be unsatisfactory. The videos lacked cultural sensitivity and were not representative of our local Hispanic/Latino populations. Many of the Spanish language diabetes educational videos available presented content which was originally published in English and dubbed with Spanish voiceover; further, the videos delivered content at a pace that was too fast and not conducive to learning. Upon further review of available content, we identified diabetes educational videos developed by Kaiser Permanente, which were subsequently approved by our CAB.

The videos used in our study included “What is Type Two Diabetes?” “How can you Succeed with Diabetes?” “How to Test your Blood Sugar” “Enjoy Exercise with Diabetes” “How to Create a Healthy Plate” “Family Fun Brings us Together” “How do Diabetes Medications Work in the Body?” and “Insulin Keeps you Healthy.” Quizzes were created for each video to test understanding of the content and to identify gaps in knowledge. The patients were given unlimited access to the videos and quizzes during the first six months on the study. The videos and quizzes were first introduced to the patients upon home delivery of the tablet and peripherals.

During the first telephone follow-up (post enrollment), the videos and quizzes were reintroduced to the patients by the research nurse. Patients were encouraged to watch the videos throughout the study. The video content and quiz answers were incorporated in the weekly diabetes educational topics for discussion.

## Teach-back method/reinforcement of key concepts

The teach-back method was used in this study to assess understanding of all education provided to patients. Education was delivered by using simple and culturally congruent words that made sense to our patients in their preferred language. We avoided information overload by discussing one key topic at a time such as signs, symptoms, and prevention of hypoglycemia followed by assessing their level of understanding by having them teach-back what they had learned.

It was important to individualize an education strategy that catered to each individual patient due to varying rates of diabetes awareness among patients, taking into consideration that patients learn and retain information at different rates. We learned that although some patients were immediately able to teach back the information provided during a telehealth visit, repetition was important, as some patients did not remember the information when discussed during subsequent visits. Specifically, we found that it was important to repeat information on HbA1c, hypoglycemia, and blood glucose monitoring (blood glucose meter and lancing device).

## Weekly topics for discussion

Weekly telehealth visits allowed us to assess patient involvement, motivation, and nonverbal communication which helped to guide the topics discussed while acknowledging patient concerns and priorities. Although we planned weekly diabetes management educational topics for discussion such as defining T2DM, HbA1c, self-monitoring of blood glucose, hyperglycemia, hypoglycemia, diabetes medications, diet, preparing for sick days, physical activity, measurements for the week (blood glucose, blood pressure, and weight), among others, it was important to consider individual patient preferences to guide the discussion.

We also found that patient collaboration and the ability to understand and retain information may be hindered by stressors. For example, during a telehealth visit, a patient was noted to be less engaged than usual, and when specifically asked “how they was feeling?” and “what was most important for them at that time?” the patient reported that it was important to have the telehealth visit, however she was grieving the loss of her loved ones, and on that day she preferred to focus our discussion on that topic. Approximately one year prior, the patient lost her father and two siblings, only four months apart, from complications related to COVID-19. As their death anniversary was approaching, her priority was to discuss concerns she had regarding their deaths, stressors, and coping strategies.

Although initially the research nurse had a planned topic, the discussion was changed and guided by patient feedback. The goal was to provide all essential resources and to address the patient’s concerns. Based on adherence data and patient feedback, we found that listening to patients and addressing their specific needs during each weekly telehealth visit; empowered, encouraged, and motivated patients to adhere to healthy lifestyle changes, self-monitoring of blood glucose (SMBG), and weekly telehealth visits despite significant stressors.

## Cultural awareness

The ability for the clinician to speak Spanish was imperative when communicating with patients in our study; however, awareness of differences in diverse Hispanic/Latino backgrounds was also critical. Our service area (the New York Metropolitan area) has a very diverse mix of patients with Hispanic/Latino heritage, and the terms and customs vary between countries of origin, sometimes even for regions within countries.

For example, our patients from El Salvador (Central America) regularly reported eating “tortillas and pupusas” whereas our patients from Colombia (South America) reported eating “arepa and pan de bono.” In addition, our patients from Puerto Rico refer to orange juice as “jugo de china” and rarely as “jugo de naranja” whereas our patients from Central America refer to it as “jugo de naranja” and rarely “jugo de china”.

Understanding common terms and phrases is important, as using relevant and commonly used terms when engaging with diverse Hispanic/Latinos has the potential to increase adherence and improve outcomes. Our study population listed sixteen different countries as their “country of origin”.

### Limitations

This study had several limitations. We conducted this study beginning in late 2019, and soon after, the pandemic began, so from March 2020 to June 2020, we could not enroll new patients. The patients on the study experienced the pandemic in a very hard hit area and were already part of an underserved population. Several patients lost family members. Additionally, we had to limit any in-person interactions with patients and switch to enrolling by telephone, which had a lower rate of acceptance. We were also forced to conduct all CAB meetings and most of our research meetings on line. We were able to arrange home visits to collect HbA1c values when patients did not want to visit clinics, but some patients may have missed their visits due to a reasonable fear of having someone come to their home during uncertain times.

## Discussion

Evidence-based culturally congruent interventions have the potential to improve patient-centered outcomes in underserved Hispanic/Latino peoples living with T2DM. As the prevalence of T2DM among this population continues to rise, implementing effective interventions is a leading priority. According to the (21); the total estimated cost of diagnosed diabetes in 2017 was $327 billion, furthermore patients with diabetes, on average have medical costs approximately 2.3 times higher compared to patients without diabetes. This emphasizes the urgent need to implement evidence-based interventions to complement the traditional standards of medical care in Diabetes to improve access to health care services and patient outcomes especially for underserved Hispanic/Latino peoples living with T2DM.

The proposed intervention was rigorously reviewed by the study team and CAB to ensure that every detail was feasible and accessible to meet the specific needs of our target population. Our individualized approach, building rapport, allowing each patient to learn at their own pace, and setting realistic goals, helped us tailor the intervention in order to accommodate as many patients as possible. The authors believe that this study has significant implications, especially considering the large population of Hispanic/Latino people in the US.

The underserved patients who participated in this study did show several overall improvements which we believe can be replicated, however, this study took place during a unique time in history – a pandemic in which medical access was scarce. It is unclear whether video-based telemanagement will continue to be an even more essential part of medical care, or if it will become obsolete due to patient preferences or new technology. Additionally, smaller health systems might not easily accommodate non-English speakers and patients who need night and weekend appointments.

This study showed improvement in patient outcomes, including a significant reduction in HbA1c in the DTM arm. This is an important finding – especially since all diabetes patients were included, not just those with uncontrolled diabetes, which is common methodology in studies looking at HbA1c. Additionally, this study looked at a rarely studied population, which we hope is studied more often in the future, in a culturally sensitive, appropriate way. Although this intervention showed highly promising results, adherence to key elements of program design, and openness to advice from the community are imperative to ensure replication, validity, and reliability. Future studies are needed to see if similar populations show similar results in different settings.

## Data availability statement

The original contributions presented in the study are included in the article/supplementary material. Further inquiries can be directed to the corresponding author.

## Ethics statement

The studies involving humans were approved by Northwell Health Institutional Review Board. The studies were conducted in accordance with the local legislation and institutional requirements. The participants provided their written informed consent to participate in this study.

## Author contributions

SM: Conceptualization, Methodology, Project administration, Validation, Writing – original draft. CN: Data curation, Writing – original draft. MW: Project administration, Writing – review & editing. VP: Funding acquisition, Project administration, Writing – review & editing. PB: Project administration, Writing – review & editing. VC: Data curation, Writing – review & editing. JM: Project administration, Writing – review & editing. NG: Data curation, Writing – review & editing. EC: Data curation, Writing – review & editing. DG: Conceptualization, Writing – review & editing. LM: Conceptualization, Writing – review & editing. YH: Conceptualization, Writing – review & editing. AM: Conceptualization, Writing – review & editing. JG: Methodology, Writing – review & editing. AM: Methodology, Writing – review & editing. SM: Writing – review & editing. RZ: Writing – review & editing. MP: Writing – review & editing. CS: Writing – review & editing. ML: Conceptualization, Writing – review & editing. MK: Writing – review & editing. RD: Writing – review & editing. RP: Conceptualization, Funding acquisition, Methodology, Project administration, Supervision, Writing – original draft.
